# County-level associations between food retailer availability and violent crime rate

**DOI:** 10.1186/s12889-022-14415-y

**Published:** 2022-11-01

**Authors:** Chelsea R. Singleton, Fikriyah Winata, Ashley M. Adams, Sara L. McLafferty, Karen M. Sheehan, Shannon N. Zenk

**Affiliations:** 1 Department of Social, Behavioral, and Population Sciences, Tulane School of Public Health & Tropical Medicine, 1440 Canal St., Suite 2200-20, New Orleans, LA 70112 USA; 2 Department of Geosciences, Mississippi State University, 355 Lee Blvd, 108 Hilbun Hall, Starkville, MS 39762 USA; 3 Department of Kinesiology and Community Health, College of Applied Health Sciences University of Illinois Urbana-Champaign, 1206 S Fourth St., Champaign, IL 61820 USA; 4 Department of Geography and Geographic Information Science, University of Illinois Urbana-Champaign, 1301 W Green St., Urbana, IL 61801 USA; 5 Departments of Pediatrics, Medical Education, and Preventive Medicine, Northwestern University, Ann and Robert H. Lurie Children’s Hospital of Chicago, 225 E Chicago Ave., Chicago, IL 60611 USA; 6 National Institute of Nursing Research, National Institutes of Health, 31 Center Dr., Bethesda, MD 20892 USA

**Keywords:** Violent crime, Retail food environment, Grocery store, Fast food, Farmers’ market

## Abstract

**Background:**

Violent crime (i.e., homicide, armed robbery, aggravated assault, and rape) continues to be a major public health concern in America. Several studies have linked the availability and density of specific features of the retail food environment, such as convenience stores and liquor stores, to violent crime rates due to the criminal activity that often occurs in and near these retailers. Nevertheless, there continues to be limited understanding of how other features (e.g., grocery stores, supercenters, restaurants, etc.) are associated with violent crime occurrence. This study aimed to fill this gap in knowledge by examining U.S. county-level associations between food retailer availability and violent crime rate.

**Methods:**

We analyzed 2014 data on 3108 counties from the U.S. Department of Agriculture’s Food Environment Atlas and Department of Justice’s Unified Crime Reporting Program. Per capita food retailer measures represented the number of stores per 10,000 county residents. Violent crime rate represented the number of police reported violent crimes per 10,000 county residents. We used spatial lag regression models to assess associations between per capita retailer availability and violent crime rate after adjusting for potential confounders (e.g., % under 18, % Black, % Hispanic, % poverty, population density, etc.). In addition, we examined stratified OLS regression models to evaluate associations by metropolitan county status.

**Results:**

Adjusted spatial regression models revealed that greater supercenter availability [β: 2.42; 95% CI: 0.91–3.93; *p*-value: 0.001] and greater fast food restaurant availability [β: 0.30; 95% CI: 0.18–0.42; *p*-value: < 0.001] were associated with higher violent crime rate.

Greater availability of farmers’ markets [β: -0.42; 95% CI: -0.77 – − 0.07); *p*-value: 0.02] was associated with lower violent crime rate. Associations varied between metropolitan and non-metropolitan counties. Stratified OLS models revealed that greater grocery store availability was associated with lower violent crime rate among metropolitan counties only. Greater fast food restaurant availability was associated with lower violent crime rate among non-metropolitan counties only.

**Conclusions:**

Certain features of the retail food environment appear to be associated with county-level violent crime rates in America. These findings highlight the need for additional research on the influence of food retail and food landscape on violent crime occurrence at the community level.

## Background

Despite steady declines since the 1990s, violent crime (i.e., homicide, rape, aggravated assault, armed robbery) continues to be a major public health issue in America [1, 2]. Violent crime is a social determinant of health and predictor of several adverse health outcomes [3, 4]. Prior studies have reported that residing in a community with high violent crime rates increases an individual’s risk for depression, substance abuse, physical inactivity, and obesity [4–7]. Historically underserved and low-resourced communities often have disproportionately higher violent crime rates, which makes residents of these communities vulnerable to crime-related health outcomes [8, 9]. Identifying the social, political, and environmental factors that drive violent crime occurrence in America is necessary to achieve equitable health outcomes.

Experts have hypothesized that the retail food environment is a structural driver of violent crime occurrence at the community level [10, 11]. There is a growing body of evidence that suggests certain food retailers significantly influence a community’s violent crime rate. For example, studies have linked greater availability and density of retailers traditionally labeled as unhealthy, such as fast food restaurants, convenience stores, and liquor stores, with higher violent crime rates [12–15]. These studies reported that violent crimes often occur in or within close proximity to these particular retailers [12–15]. Furthermore, emerging evidence suggests that areas with several large discount retailers, such as supercenters (e.g., Wal-Mart) and dollar stores, experience more crime [16].

The premise that greater availability of certain food retailers can increase crime rates aligns well with the Routine Activity Theory proposed by Cohen and Felson in 1979 [17]. The theory postulates that crime occurs when a motivated offender meets a suitable target in the absence of a capable guardian or deterrent [17]. Food retailers may be crime attractors because they *1)* carry relatively large amounts of money due to the high volume of cash transactions, *2)* stock commodities of great personal and financial interest to people (e.g., food, alcohol) and *3)* often have minimal security [17, 18]. This theory coupled with findings from prior studies suggest that the landscape of a community’s retail food environment may play an important role in crime occurrence.

Despite the existing evidence on the relationship between food retailer availability and violent crime rate, there are some key gaps in knowledge. Most prior studies solely examined unhealthy food retailers and their spatial relationship with crime in one specific region or city [12–15]. Furthermore, most prior studies focused on the urban context and did not provide insight into the these associations in rural settings [12–15, 18]. The objective of this study is to fill these gaps in knowledge by examining U.S. county-level associations between violent crime rate and the availability of retailers that are traditionally labeled as healthy (e.g., grocery stores, supercenters, and farmers’ markets) and unhealthy (e.g., fast food restaurants and convenience stores) in regards to their food and beverage offerings. In addition, this study will evaluate the strength and significance of these associations among metropolitan and non-metropolitan counties separately. Findings from this research will contribute much needed knowledge to the field on the role of the retail food environment in violent crime occurrence. Given findings from existing literature, we hypothesize that greater availability of convenience stores and fast food restaurants will be associated with a higher violent crime rate.

## Methods

### Data sources

We obtained 2014 data on 3108 counties (and equivalents) within the contiguous United States from two public sources: The U.S. Department of Agriculture (USDA) Food Environment Atlas and the United States Department of Justice (DOJ) Uniform Crime Reporting Program [19, 20]. We excluded counties in Alaska and Hawaii due to missing food environment data. The USDA’s Food Environment Atlas contains 2014 data on per capita food retailer availability and socio-demographics for all counties [19]. The DOJ Unified Crime Reporting Program collects and reports county-level data on violent and non-violent crime events each year [20]. The DOJ dataset we obtained via the Inter-University Consortium for Political and Social Research was missing violent crime measures for Illinois and Florida. To complete the violent crime data, we accessed publicly available 2014 data from the Illinois State Police Uniform Crime Reporting Program [21]. Furthermore, we retrieved 2014 data from the Florida Department of Law Enforcement to fill in the missing values for Florida [22]. The institutional review board at The University of Illinois at Urbana-Champaign deemed this exempt research.

### Measures

**Fig. 1:**
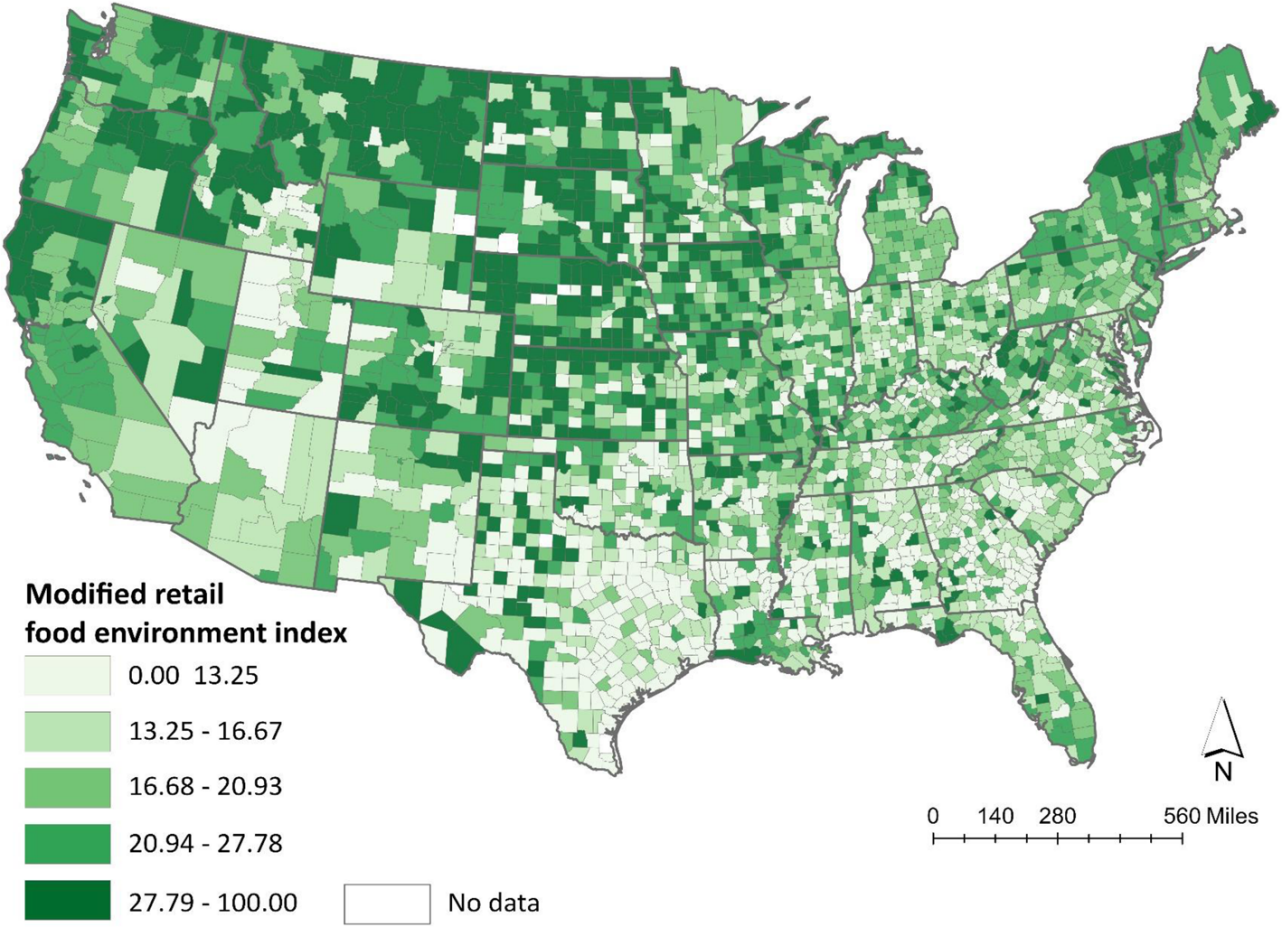
Distribution of the CDC’s Modified Food Retail Environment Index by U.S. County in 2014. The data presented in this map are from the U.S. Department of Agriculture’s Food Environment Atlas. The Center for Disease Control and Prevention’s (CDC) Modified Food Retail Environment Index represent the proportion of food retailers in the county that are healthy retailers (e.g., grocery stores, supermarkets, supercenters, farmer’s markets). A larger index score out of 100 suggests a healthier retail food environment. Categories represent quartiles for the index score

We examined the following per capita measures from the USDA’s Food Environment Atlas: grocery stores, supercenters, convenience stores, fast food restaurants, full-service restaurants, and farmers’ markets. Per capita represents the total number of retailers available per 10,000 county residents. All measures reflect 2014 estimates with the exception of farmers’ markets which reflect 2016. Data on farmers’ market availability were not available for 2014. Definitions of food retailers align with the North American Industry Classification System (NAICS) [19]. Grocery stores, which include supermarkets, are establishments that offer a complete line of staple foods (e.g., fruit, vegetables, bread, meat, and dairy). Supercenters, which include warehouse club stores (e.g., Costco), sell a complete line of groceries in addition to items such as clothing, furniture, and household appliances. Convenience stores are establishments that sell a limited line of staple foods. NAICS classifies gas stations as convenience stores. Fast food restaurants are outlets that provide ready-to-eat food to customers who generally pay before eating. Full-service restaurants offer waiter service and allow customers to pay after eating. Farmers’ markets are outlets in which two or more vendors sell agricultural products directly to consumers at the same location and time. We applied the CDC’s Retail Food Environment Index (mRFEI) to these data to assess the overall healthfulness of each county’s retail food environment [23]. The mRFEI represents the percentage of all food retailers in a specific geographic area that are healthy (i.e., grocery stores, supercenters, farmers’ markets) [23]. When comparing counties, the county with a higher score out of the maximum 100 points indicates that more healthy food retailers are available. Figure 1 displays the 2014 mRFEI scores for counties located in the contiguous United States.

**Fig. 2:**
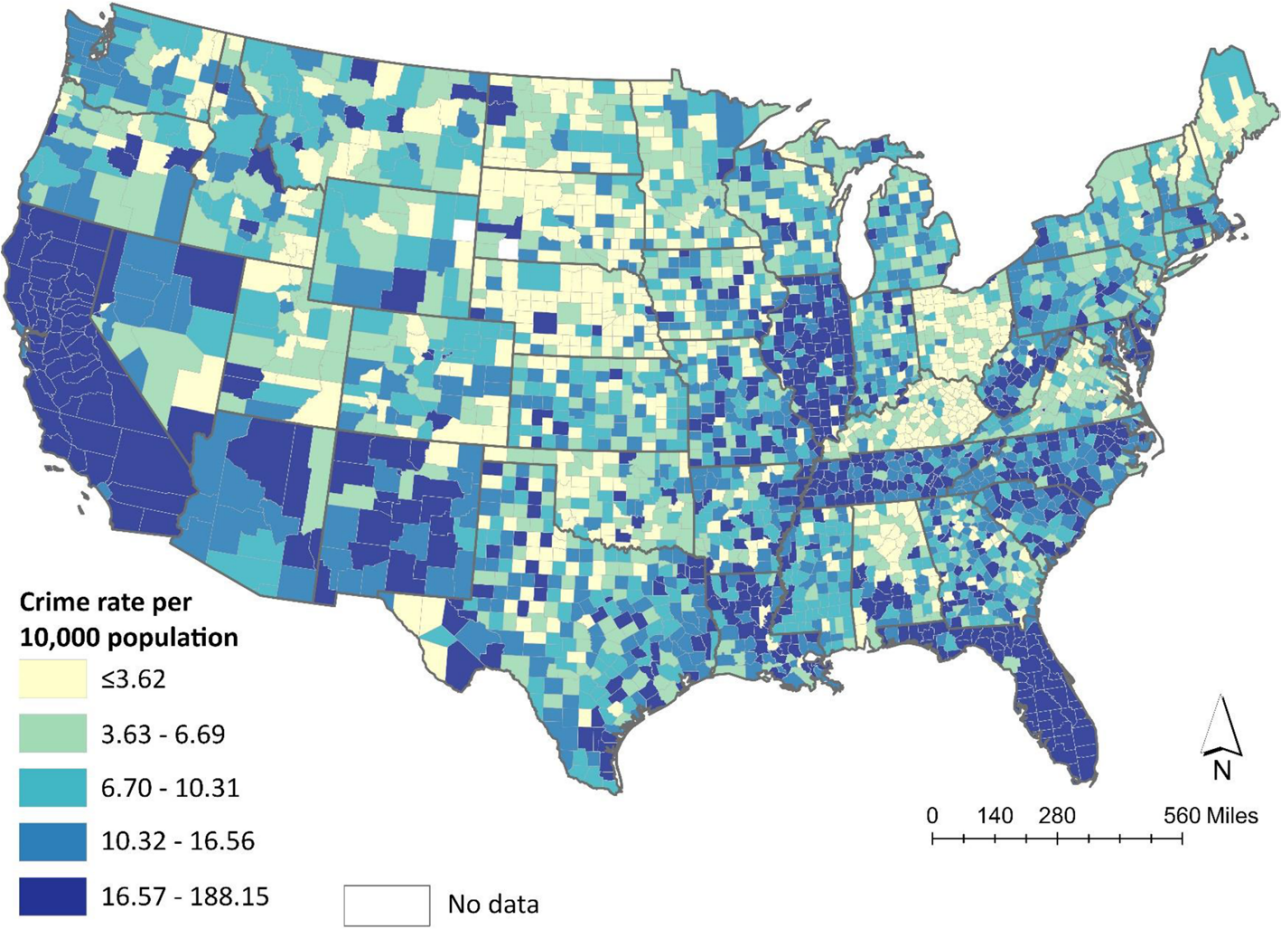
Distribution of Violent Crime Rate by U.S. County in 2014. The data presented in this map are from the U.S. Department of Justice’s Uniform Crime Reporting Program. Data from Florida and Illinois are from the Florida Department of Law Enforcement and the Illinois State Police Uniform Crime Reporting Program, respectively. Violent crime rate represents that total number of homicides, rapes, armed robberies, and aggravated assaults per 10,000 county residents. Categories represent quartiles for violent crime rate

We calculated each county’s violent crime rate for 2014 using the FBI’s definition of violent crime, which includes homicide, rape, aggravated assault, and armed robbery [24]. Total violent crime rate reflects the number of FBI designated violent crimes that occurred per 10,000 county residents. Figure 2 displays the number of violent crimes per 10,000 population in the contiguous United States. Socio-demographic measures examined include % under 18, % non-Hispanic Black, % Hispanic, % living below the federal poverty threshold, annual median household income, % residents who are low-income and have low-access to a grocery store, population density and metropolitan county status. All measures reflect 2010 estimates from the U.S. Census Bureau [19]. For the % low-income and low-access measure, the USDA defined low-income as having an annual family income ≤200% of the federal poverty threshold. They defined low-access as living more than 1 mile from a grocery store in urban areas and 10 miles in rural areas [19]. Population density reflects the number of people per square mile [19]. According to the USDA, a metropolitan (henceforth: metro) county is a county with ≥50,000 residents or is outlying and economically tied to a county with a large high-density urban area [19]. They consider all other counties to be non-metropolitan.

### Data analysis

Since spatial dependency may appear at the county-level for our measures of interest, we used spatial and statistical analysis methods to examine associations between food retailer availability and violent crime rate. All analyses were performed with GeoDa and R software [25, 26]. All maps were created using ArcGIS Pro 2.8 [27].. We calculated descriptive statistics (i.e., median and range) for all measures of interest among all counties and stratified by metro county status. The Mann-Whitney Wilcoxon Test was used to determine if county-level characteristics for socio-demographics, food retailer availability, and violent crime rate differed between metro and non-metro counties [28].

We ran OLS regression models and assessed the Lagrange Multiplier Test results to determine the most appropriate spatial regression models (spatial lag model vs. spatial error model). The OLS regression models included the food retailer measures and all covariates: % under 18, % non-Hispanic Black, % Hispanic, % poverty, population density, and metro county status. The spatial lag model considers the spatial dependency of dependent variables in one area with other areas; the spatial error model considers the dependency of error values of an area with errors in other areas [29]. For this analysis, the Lagrange Multiplier Test revealed significant *p*-values for both the spatial lag and error models. However, the robust test indicated a smaller *p*-value for the spatial lag model. Thus, we used spatial lag models to examine associations between food retailer availability and violent crime rate while controlling for covariates. Each per capita food retailer measure was included, separately, in a spatial lag model with the covariates, and we used Queen contiguity for spatial weight in all models. The measure for mRFEI was assessed in a separate model.

To evaluate relationships between food retailer availability and violent crime rate among metro and non-metro counties independently, we examined stratified non-spatial OLS regression models. We assessed non-spatial OLS models since U.S. counties are not adjoined by metro county status. Non-spatial OLS models were also adjusted for covariates. We reported the parameter estimates (β), 95% confidence intervals (CI), and *p*-values from all models. *P*-values less than 0.05 were considered statistically significant.

## Results

County-level characteristics are presented in Table 1 among all counties and stratified by metro county status. The percentage of metro and non-metro counties were 37.3 and 62.7%, respectively. We observed significant socio-demographic differences between metro and non-metro counties. The median percentages of non-Hispanic Black (5.3%) and Hispanic (4.5%) residents were higher in metro counties compared to non-metro counties (0.8 and 2.6%, respectively). Comparatively, the median percentage of impoverished residents was higher (16.8%) and the annual median household income was lower ($38,604) among non-metro counties compared to metro counties (14.3% and $46,985, respectively). Median per capita food retailer estimates were comparable between metro and non-metro counties, and median violent crime rate was similar between metro and non-metro counties.

**Table 1:** Characteristics of U.S. Counties in 2014 by Metro County Status, Median (Minimum-Maximum)

Characteristic:	All Counties *N* = 3108	Metro Counties 1159 (37.29)	Non-Metro Counties 1949 (62.71)	***P***-value^**a**^
*Socio-Demographics*				
% Non-Hispanic Black	1.92 (0–85.44)	5.31 (0–78.41)	0.82 (0–85.44)	< 0.001
% Hispanic	3.28 (0–95.74)	4.46 (0.35–95.74)	2.59 (0–95.68)	< 0.001
% Poverty	15.90 (0–50.10)	14.30 (0–41.30)	16.80 (0–50.10)	< 0.001
Median Household Income, $	41,194 (0–119,075)	46,985 (26,993 – 119,075)	38,604 (0–105,987)	< 0.001
% Low Access & Low Income	6.14 (0–72.27)	6.11 (0–72.27)	6.17 (0–59.98)	0.58
Population density, pop/sq. mile	44.47 (0–62,430.70)	51.63 (0.29–62,430.70)	41.47 (0–44.013.77)	< 0.001
*Food Retailer Availability* ^*b*^				
Grocery Stores	1.94 (0–25.45)	1.95 (0–25.45)	1.93 (0–22.08)	0.77
Supercenters	0.14 (0–2.48)	0.15 (0–2.48)	0.14 (0–2.11)	0.22
Convenience Stores	5.46 (0–46.01)	5.29 (0–38.17)	5.56 (0–46.01)	0.007
Fast Food Restaurants	5.79 (0–55.56)	5.79 (0–35.13)	5.79 (0–55.56)	0.38
Full-Service Restaurants	6.76 (0–138.89)	6.80 (0–76.34)	6.72 (0–138.89)	0.06
Farmers’ Markets^*c*^	0.30 (0–14.45)	0.29 (0–9.10)	0.3 (0–14.45)	0.89
mRFEI Score	18.39 (0–100)	18.38 (0–100)	18.41 (0–100)	0.82
*Violent Crime Rate* ^*d*^	8.43 (0–188.15)	8.65 (0–92.81)	8.24 (0–188.15)	0.17

*mRFEI* Modified Retail Food Environment Index

^a^*P*-values in this table were calculated using Mann-Whitney Wilcoxon tests

^b^Measures for food retailers represent availability per 10,000 county residents

^c^The measure for farmers’ markets reflects the 2016 estimate

^d^Violent crime rate represent the number of murders, rapes, aggravated assaults, and armed robberies per 10,000 county residents

Results from spatial lag models and stratified OLS models are displayed in Table 2. After adjusting for covariates, all food retailer measures were significantly associated with violent crime rate among all counties except per capita grocery stores and convenience stores. We observed an inverse association between violent crime rate and the measures per capita full-service restaurants [β: -0.07; 95% CI: − 0.13 – − 0.01; *p*-value: 0.03] and farmers’ markets [β: -0.42; 95% CI: − 0.77 – − 0.07; p-value: 0.02]; higher availability of these retailers was associated with lower violent crime rate. MRFEI score was also inversely associated with violent crime rate [β: -0.07; 95% CI: − 0.09 – − 0.05; *p*-value: < 0.001]. We observed strong positive associations between violent crime rate and the measures per capita fast food restaurants [β: 0.30; 95% CI: 0.18–0.42; *p*-value: < 0.001] and supercenters [β: 2.42; 95% CI: 0.91–3.93; *p*-value: 0.001]; higher availability of these retailers was associated with higher violent crime rate.

**Table 2:** Regression Models Examining Associations between Food Retailer Availability and Violent Crime Rate by Metro County Status in the U.S.

	All Counties^***a***^		Metro Counties^***b***^		Non-Metro Counties^***b***^	
	β (95% CI)	*P* value	β (95% CI)	*P* value	β (95% CI)	*P* value
*Food Retailer:*						
Grocery Stores	-0.07 (−0.27, 0.13)	0.43	-0.45 (−0.88, − 0.02)	0.03	− 0.21 (− 0.46, 0.04)	0.11
Supercenters	2.42 (0.91, 3.93)	0.001	2.62 (−0.61, 5.85)	0.11	1.97 (−0.13, 4.07)	0.06
Convenience Stores	0.07 (−0.05, 0.19)	0.23	0.27 (0.02, 0.52)	0.04	−0.05 (− 0.21, 0.11)	0.52
Fast Food Restaurants	0.30 (0.18, 0.42)	< 0.001	0.19 (−0.06, 0.44)	0.15	0.31 (0.13, 0.49)	< 0.001
Full-Service Restaurants	−0.07 (− 0.13, − 0.01)	0.03	−0.23 (− 0.39, − 0.07)	0.008	−0.06 (− 0.16, 0.04)	0.16
Farmers’ Markets^*c*^	−0.42 (− 0.77, − 0.07)	0.02	−0.88 (− 1.76, 0.002)	0.05	− 0.76 (− 1.23, − 0.29)	0.001
*Food Environment Index:*						
mRFEI Score	−0.07 (−0.09, − 0.05)	< 0.001	− 0.15 (− 0.21, − 0.09)	< 0.001	−0.10 (− 0.14, − 0.06)	< 0.001

*mRFEI* Modified Retail Food Environment Index, *SE* standard error, *CI: 95%* confident interval

^a^ Spatial lag regression models adjusted for % Non-Hispanic Black, % Hispanic, % poverty, population density, and metro county status

^b^OLS regression models adjusted for % Non-Hispanic Black, % Hispanic, population density, and % poverty

^c^The measure for farmers’ markets reflects the 2016 estimate

Stratified models identified associations between food retailer availability and violent crime rate among metro and non-metro counties, separately. For both metro and non-metro counties, higher per capita farmers’ markets and mRFEI were associated with a lower violent crime rate. Greater per capita grocery stores [β: -0.45; 95% CI: − 0.88 – − 0.02; *p*-value: 0.03] and full-service restaurants [β: -0.23; 95% CI: − 0.39 – − 0.07; *p*-value: 0.008] were associated with lower violent crime rate among metro counties only. Greater per capita convenience stores [β: 0.27; 95% CI: 0.02–0.01; *p*-value: 0.04] was associated with a higher violent crime rate among metro counties only while greater per capita fast food restaurants [β: 0.31; 95% CI: 0.13–0.49; *p*-value: < 0.001] was associated with a higher violent crime rate among non-metro counties only.

## Discussion

This study aimed to evaluate the relationship between the food retailer availability and violent crime rate at the county level. While our analyses linked the availability of several food retailers to violent crime rate among all U.S. counties, some of these associations were not significant among metro and/or non-metro counties. This suggests that certain food retailers may be more relevant to violent crime occurrence in urban areas compared to rural areas and vice versa. Prior research has documented significant differences in food retailer availability and crime rates between urban and rural areas of America [30–33]. These differences may explain, in part, the differences between metro and non-metro counties observed in our study. Some of our study findings align well with prior research while some do not. Below we discuss the implications of our findings by retailer category: unhealthy vs. healthy.

### Unhealthy retailers

Current evidence indicates that greater availability of food retailers traditionally labeled as unhealthy (e.g., fast food restaurants and convenience stores) is associated with higher violent crime rates [12–15, 18]. We found that fast food restaurant availability was associated with a higher violent crime rate among all counties (pooled) and non-metro counties only. Although the parameter estimate for fast food retailers among metro counties was positive (0.19), it did not achieve statistical significance. Among non-metro counties, each additional fast food retailer was associated with an additional 0.31 crime incidents per 10,000 persons. This is estimate is concerning given the volume and steady growth of fast food outlets in the U.S. in the prior decade [13]. Many studies on fast food availability and crime have focused solely on urban cities. Askey et al. [13] reported that greater availability of fast food restaurants in Seattle, WA was associated with higher crime counts. Bernasco and Block [18] documented a significant association between number of fast food restaurants and robbery counts at the census-tract level in Chicago, IL. To our knowledge, there is no available literature of the relationship between fast food availability and crime in rural areas. This information is needed to provide more context to urban-rural differences in the influence of fast food availability on violent crime.

Like previous studies, we found that greater convenience store availability is associated with a higher violent crime rate, particularly in metro counties [12–15]. Each additional convenience store was associated with an 0.27 additional crime incidents per 10,000 persons in metro counties. The availability and density of convenience stores is often high in large urban centers [12]; low-income communities and communities of color in urban areas are more likely to have an overabundance of these stores [14]. Thus, having a large amount of convenience stores may have important implications for violent crime occurrence in urban communities. Unfortunately, the heterogeneity of food stores that counted toward the convenience store definition made it impossible to evaluate differences by convenience store type (e.g., gas station, liquor store, pharmacy). However, other studies have linked the availability and density of specifically corner stores and liquor stores to crime in urban areas [12–15]. Furr-Holden et al. [14] concluded that violent crime events were more prevalent near corner stores in Baltimore, MD compared to drug treatment centers. Jennings et al. [15] reported that more violent crime events occurred in neighborhoods with greater availability of alcohol retailers. The scientific evidence linking convenience store availability to crime in urban areas is the most robust and consistent in this space.

### Healthy retailers

For food retailers traditionally labeled as healthy (e.g., grocery stores, supercenters, farmers’ markets), our analyses identified positive and negative associations with violent crime at the county level. A key finding from our study is the positive relationship between supercenter availability and violent crime rate. Greater supercenter availability was associated with higher violent crime rate among all counties (pooled). Every additional supercenter was associated with, on average, 2–3 additional violent crime incidents per 10,000 persons. The number of supercenters per population in counties is not as high as convenience stores or fast food retailers due to the size of these retailers. However, the total amount of supercenters in the U.S. has increased exponentially in the last 20 years [16]. Thus, simply introducing a supercenter has implications for crime occurrence. Wolfe and Pyrooz [16] reported a direct relationship between opening a supercenter, specifically Wal-Mart, and crime occurrence. Their findings also documented an increase in crime over time in counties that recently received a Wal-Mart [16]. Grey literature has discussed the impact of large discount stores, such as supercenters, on violent and non-violent crime occurrence [34]. While these data suggest that supercenters are crime attractors, it is possible that these stores are more likely to target and open in low to middle-income areas that already have high crime rates. Since supercenters are highly prevalent in the U.S. and serve as major food sources for urban and rural populations, this is an important topic that warrants further research [35].

MRFEI score and farmers’ market availability were negatively associated with violent crime rate among both metro and non-metro counties; the magnitude of the associations were similar between metro and non-metro counties. The parameter estimates for mRFEI score were small (− 0.15 to − 0.07), which suggests a single unit increase in mRFEI score (scale: 0–100) is associated with a relatively small decrease in violent crime rate. On the other hand, the parameter estimates for farmer’s market availability were larger in magnitude. Each additional farmers’ market was associated with 0.42 to 0.88 fewer crime incidents per 10,000 persons. Grocery store availability was negatively associated with violent crime rate among metro counties only; each additional grocery store was associated with 0.45 fewer crime incidents per 10,000 persons.

In general, current evidence on the availability of food retailers traditionally labeled as healthy and violent crime occurrence is limited and unclear [18, 36]. While findings from this study suggest that greater availability of healthy food retailers, particularly grocery stores, is associated with less violent crime, other studies do not fully support this hypothesis [18, 36]. Rabinowitz [36] found that greater access to supermarkets was associated with more criminal activity in Connecticut. Furthermore, Bernasco and Block (2011) found that the number of robberies in Chicago, IL at the block level increased with increasing proximity to a grocery store [18]. To our knowledge, there is no available evidence that can provide us additional insight to the relationship between violent crime and retailers such as farmers’ markets. These findings highlight the need for additional research on the relationship between availability of these particular retailers and crime.

### Limitations

This research has limitations. Because the data were cross-sectional, we were unable to explore longitudinal relationships between food retailer availability and violent crime rate. Although data on violent crime was available for several years, the food environment data was not available. The data sources only offered county-level information on retailer availability and violent crime rate. Counties differ greatly in geographic and population size. Relying on county-level data limited our ability to evaluate smaller geographic units (e.g., census tracts or blocks) and use spatial regression modeling to examine the importance of food retailer density. Future research on this topic should utilized a smaller geographic unit. In a smaller unit analysis, researchers may incorporate network data (e.g., roads, public transportation) and other community amenities. Furthermore, it will permit a proper assessment of disparities in access to healthy and unhealthy food retailers between communities.

As previously mentioned, we acquired violent crime data for Florida and Illinois from sources other than the DOJ Unified Crime Reporting Program [21, 22]. Although we performed quality checks to ensure the data collection approaches and definitions for violent crimes were similar, differences in reporting protocols may have affected the data for those two states. The measure we used for farmers’ market availability reflects the 2016 estimate rather than 2014. It is possible that the availability of farmers’ markets is some U.S. counties changed between these years. The analyses performed utilized several statistical tests, which increased chance of Type I error. Lastly, this was an ecological evaluation, so we did not examine individual-level factors (i.e., food shopping behaviors) in relation to violent crime rate. All conclusions should be drawn at the county level.

Given the nature of this analysis, we cannot rule out reverse causality. Most studies on this topic have hypothesized that high availability and density of certain food retailers can influence crime rate. However, recent studies have hinted at a possible bi-directional relationship between food retailer availability and crime [37, 38]. For example, Mui et al. [38] found that crime rate in Baltimore, MD was associated with a greater decline in the healthfulness of the retail food environment over time. It is possible that violent crime can adversely affect the retail food environment by causing premature closure of retailers or by discouraging specific retailers from opening in a community [37]. High crime rates may deter customers from using a food retailer leading to low profitability and high risk of closure [39, 40]. Given the complexity of these pathways, additional mixed-methods research involving multi-dimensional and longitudinal data is needed to determine how the retail food environment is associated with crime.

## Conclusions

We cross-sectionally linked the availability of several food retailers to violent crime rate at the county level and found significant differences by metro county status. These findings may have important implications for policies and interventions that focus on violent crime prevention or food retail expansion in America. If the retail food environment is a significant structural driver of violent crime occurrence, future initiatives may have to consider the overall design of a community’s food landscape or make appropriate changes that will address crime occurrence. Nevertheless, there is an ongoing need for more research on the relationship between food retail and crime. Additional research that produces stronger empirical evidence would greatly strengthen the knowledge base. Future studies should expand this line of research to address major inequities in nutrition and crime and exist at the community level in America.

## Data Availability

All data used in this analysis are publically available. The USDA provided the county-level food environment and socio-demographic data: https://www.ers.usda.gov/data-products/food-environment-atlas/. County-level violent crime data was collected by the U.S. Department of Justice’s Uniform Crime Reporting Program but obtained from the Inter-University Consortium for Political and Social Research: https://www.icpsr.umich.edu/web/ICPSR/studies/36399.

## Abbreviations

CDC: Centers for Disease Control and Prevention
DOJ: Department of Justice
mRFEI: Modified Retail Food Environment Index
NAICS: North American Industry Classification System
USDA: United States Department of Agriculture
